# Influence of digitalization on political participation among young adults in contemporary Europe

**DOI:** 10.12688/openreseurope.21521.1

**Published:** 2025-11-26

**Authors:** Yusuf Abubakar WARA

**Affiliations:** 1 International Relations, KASTAMONU UNIVERSITY, Kastamonu, Kastamonu, 37150, Turkey

**Keywords:** Europe, Digitalization, Political Participation, Youth, Meta-synthesis, E-democracy

## Abstract

**Background:**

Digital Politics is considered one of the tremendous factors of improving political participation and encouraging civic duties among youth across Europe. This study examines how digitalization has transformed political participation among young adults in contemporary Europe.

**Method:**

Through a meta-synthesis of 30 scholarly articles published between 2014 and 2025 on the Web of Science database and relevant sites, the study adopts a systematic qualitative meta-synthesis design to assess how digitalization influences political participation among young adults in contemporary Europe. Drawing on mobilization theory, it explores how digital technologies such as e-democracy, e-governance, and social media platforms have redefined youth political engagement, lowering barriers to participation and fostering new forms of civic activism.

**Results:**

The results show that digitalization has expanded political arenas beyond traditional structures, creating accessible and decentralized spaces for expression, mobilization, and collective action. Movements such as Fridays for Future illustrate how online activism translates into transnational political engagement. However, the study also identifies significant challenges associated with digital politics. Persistent digital divides, disparities in technological literacy, and unequal access to infrastructure hinder equitable participation across Europe. Furthermore, cyber insecurity, misinformation, and slacktivism undermine the transformative potential of digital politics by fostering polarization and reducing the depth of civic commitment. Despite these challenges, digitalization remains a catalyst for participatory innovation, particularly when coupled with media literacy initiatives and inclusive digital governance frameworks.

**Conclusion:**

The study concludes that a sustainable digital democracy requires cross-sectoral collaboration between governments, civil society, and educational institutions to promote digital equity, enhance accountability, and counter polarization. By integrating findings from across Europe, this research contributes to understanding how digital transformation simultaneously empowers and constrains democratic participation in the 21st century.

## Plain language summary

This article is about the influence of technological tools such as social media on increasing the youth political participation in modern Europe. The paper indicates that most young people across the continent participate in e-voting, online petition and even online protests to send messages to the leaders regarding events that they think need urgent attention. The article argues that though the digital world has increases youth political participation, it also creates digital divides as some people may not have equal access to modern technological gadget and internet, it also creates increase in the spread of disinformation and fake news. In order to address the problems associated with digital politics, this research recommends infrastructural development and ensuring unified digital literacy programs at an affordable rate to the least developed parts of communities across the continent. Civil societies should also partner with the government in a collaborative effort to ensure gender equity and assist people with disability and create a fair and inclusive society that would address all disparities, thereby promoting universal digital equity in all communities.

## Introduction

The older cohorts across Europe have historically shown a higher participation in traditional politics than young adults ( Sloam, 2014: 2019). This has raised concerns and created an atmosphere of deliberation on why youth incline to lower political participation, especially in the continent's mainstream politics ( Briggs, 2017: 63–9). With digitalization, the notion is changing. In other words, in the traditional political activities, Youth participation in electoral politics is diminishing, while engagement in other kinds of participation is rising, resulting in a more diverse array of political actions among young people compared to earlier generations. However, the increased digitalization of several societal sectors over the past two decades has led to a dynamic arena of political participation adopted by youth in the contemporary European political lifestyle. For the young people who are tagged as the digital generation, the internet and social media are not just tools for communication, but also form an integral part of the social and political lifeworld of the current generation.

In modern times, the digital transformation of European societies has brought in new styles in which citizens, especially the youth, engage with politics. Digitalization has therefore redefined traditional spaces of political communication and even created an avenue of participatory arena through which social media, e-participation, and online platforms for digital natives (youth) to utilize in their day-to-day political and economic activities ( Koc-Michalska & Lilleker, 2017: 1-4). This digitalization, however, is not without challenges. Through efforts such as hashtag campaigns, it opens the door to action, makes political information more accessible, and makes it easier for people to express themselves through digital protests. As a result of the dissemination of misleading information and the growing of digital divides, the problem is that it weakens societal peace and unity ( Chagas *et al*., 2022: 42–45). So, it is important to take into consideration both the enabling and restricting aspects when trying to understand how digitization has affected the political involvement of young people in modern Europe. 

Shifting away from conventional political engagement the young adults in Europe in the past two decades have incline towards digital politics which led to the emergence of new form of political participation that outdated traditional political institutions and hierarchies of political power ( Vaccari, 2013: 25). Young Europeans choose to be active through digital political agora, where activism can be instantaneous, decentralized, and transnational in scope compared to conventional political arrangement ( Gerbaudo, 2018). This change has challenged the established monopoly of politics in society by introducing new patterns of political activities, especially among young members of a political community ( Christensen, 2011). On the other hand, the extent to which these digital forms of interaction result in long-lasting political effect is a contentious issue, which has led to the need for crucial investigations about democratic legitimacy, representation, and inclusion in the digital era. Upon this enquiry lies the idea that the influence of digitalization on youth political participation in Europe is not isolated from broader political and social structures ( Dahlgren & Alvares, 2013: 48–55). This is because digital access remains uneven across macroeconomic groups. This uneven access creates a participatory divide even in regions and educational backgrounds, justifying the series of societal inequalities. Another problem with digital space is that, despite the fact that it gives a voice to those who are marginalized in political discourse, it also puts young people at danger of being manipulated by algorithms and being trapped in echo chambers, both of which can stifle their ability to freely express themselves about political concerns ( Rea, 2022: 15). Given the increasing political polarization that is taking place, the internet has the potential to be both a force that brings people together and a force that drives them apart. As a result, we need to take a close look at how digitization presents both opportunities and threats, paying special attention to how these issues of participation intersect with those of governance, democratic resilience, and inequality.

 In this article, we will look at how digitalization has affected youth political engagement in Europe, and how those effects are complicated and often conflicting. Although the internet's early advocates viewed it as a solution to political apathy and its detractors as a catalyst for societal strife, contemporary scholarly discourse illuminates a more complex reality. This article argues that digitalization creates new political opportunities in political participation and its transformative features. However, the article also indicates that digitalization is a tool for exacerbating existing inequalities and bringing about new threats to the quality of democratic governance. This review seeks to synthesize current studies to offer a thorough picture of this transition and its consequences for European democracy.

## Background

### Literature review

The study of young adults in political participation has evolved from conventional styles limited to voting and party membership to a broader conceptualization that includes civic engagement, protest, and lifestyle politics. This broader political activity entails digitalization that can be actualized through a series of digital networks. These networks include activities such as signing online petitions and using hashtags for numerous activisms. Tsouparopoulou *et al*. (2025) have presented the attitudes of young Europeans (15–35 years) concerning digital behaviors in both political and social activities. They shed light on the youth digital civil participation in the broader social environment. They testified that countries with higher youthful digital optimism have greater digital civic engagement, suggesting a nexus between digitalization and political participation in political societies. Their analysis further highlights the connection between socio-demographic factors such as educational level, income status, and digital politics in communities. Their article's high points rest on their classification of optimistic and pessimistic attitudes toward digitalization among European young people. It was discovered by Tsouparopoulou and colleagues ( 2025: 240) that young individuals in countries such as France, Slovenia, and the Netherlands had a greater degree of skepticism regarding technology compared to those in Finland, Norway, and Spain. The findings of their research are especially interesting in that they illustrate patterns of internet usage. These patterns demonstrate that Spain and Sweden are the countries with the highest daily internet usage in Europe, in contrast to Belgium, Hungary, and Greece, which have lower averages. Overall findings reveal that Austria and Finland consistently lead in better civic involvement and digital skills among those aged 15 to 35.

In another perspective, Tiidenberg *et al*. analyze digital politics as an alternative to the continuous decline of conventional politics in Europe. They focused on how youth use social media to facilitate their political engagement, which is altered by their access to digital space, their perception of datafication and surveillance ( Tiidenberg *et al*., 2024: 355–8). The strong point of their article is their analysis of the perception and motivation of young adults' digital participation in the United Kingdom, Estonia, and Greece. The youth interviewed from the three countries see online activities as an important form of digital politics, even though some regarded them as incomparable to conventional political activities on the campaign grounds.

Despite the pessimism or the thinking of a lack of efficiency of digital politics compared to conventional politics, research on European young adults' digital political participation shows how the new alternative to politics shapes young adults' political ideas. Online activities among these new generations tend to increase expressive, contacting, and protest activities, while the effect of traditional politics, such as voters’ turnout, is decreasing and becoming more context-dependent. This draws some researchers' attention to a synthesis of the effectiveness of online and offline political participation. Oser ( 2022: 612–16) cites research showing that offline participation is more strongly linked to political success, despite the pervasiveness of digital media and the internet. However, modern European youth, more likely to participate in politics through online networks, have not bought into this notion. Research on the youth digital influence in European Parliamentary elections indicates that technological advancements play a key role in young voter participation in European political processes. In the 2004 to 2014 European parliamentary elections, youth participation was limited or did not increase. However, due to the increase in the relevance and popularity of digital political elections after 2019, there has been an increase in youth participation in European Parliamentary elections ( Gelkhauri, 2025: 14–15), indicating the role of digitalization in influencing young adult political activities.

## Method

This study adopts a systematic qualitative meta-synthesis design to assess how digitalization influences political participation among young adults in contemporary Europe. Meta-synthesis is an interpretative approach that amalgamates results from several qualitative investigations to generate novel theoretical ideas ( Nye *et al*., 2016). The method was adopted to capture the multidimentionality of digital political participation across diverse social contexts.

In terms of data collection, a thorough literature review was performed utilizing the Web of Science database. The investigation encompassed the timeframe from 2014 to 2025 and exclusively featured peer-reviewed academic publications authored in English. Search keywords encompassed combinations of digitalization, youth, political engagement, Europe, e-democracy, and online activism. The inclusion criteria mandated that each study (a) concentrate on European contexts, (b) examine digital tools or platforms concerning political engagement, and (c) encompass youth populatons aged 15–35. Out of the initual pool 90 studies, 30 articles satisfied the inclusion criteria and were chosen for comprehensive analysis.

For data analysis, we followed the Noblit and Hare’s (1998) seven-step interpretive meta-sythesis structure ( Stafford & Farshadkhah, 2020). We meticulously examined each paper and using inductive coding to discern fundamental concepts, methodologies, and results. Themes that includes digital mobilization, e-participation, digital inequality, technological empowerment, and digital activism were identified. Comparative analyses across studies were performed to synthesize these findings into overarching theme categories that represent both facilitating and inhibiting aspects of digital involvement in modern Europe.

To ensure analytic rigor of the research, the sythesis utilized triangulation by comparing interpretations from various studies. Reflexivity was maintained by recording analytical choices during the synthesis process. The objective was not to quantitatively gather data but to interpretively synthesize findings, producing a nuanced comprehension of how digitalization influences democratic participation in modern Europe.

### Mobilization theory in the electronic republic

According to Cameron ( 1974: 138), Karl Deutsch describes mobilization as a movement from traditional socioeconomic activities to a fresh framework of political socialization. This change is driven by modern occurrences such as the revolution in information technology. One of the driving forces behind this trend in digital politics is the reduction in participation expenses that is made possible by digital technology. It increases information access, thus mobilizing previously disengaged youth in new patterns of political processes ( Boulianne, 2015: 525). By engaging in social media, some scholars believe that social networks are expanded and thus create a larger digital space for political activities among the mobilized youth members of a particular community.

Mobilization theory in the context of electronic republic, which tends to reawaken the apolitical members of the community is distinguished from normalization theory that posits that digital platforms benefit the established political interest, thus instead of mobilizing the forgotten it tends to amplify the already existing participation gaps in the political environment- thus preserving the status quo ( Kim *et al.*, 2025: 65). Mobilization further differs from normalization in that it focuses on understanding the changing nature of political actors in redefining the nature of politics, which digitalization appears as a hallmark.

The theory encompasses various modern political actors and their updated activities to suit the current global technological village. In the context of digital politics, the theory has been transformed and utilized to readjust or fill the lacunas of lethargic political participation among the younger generation through mobilizing digital grassroots campaigns with the help of social media platforms ( Asimakopoulos *et al*., 2025: 18–19). Today, in Europe and elsewhere, the internet and digital communication technologies have not only lowered the cost of coordination but also motivated young adults to engage in online politics, which might not be possible offline.

## Results

### Young adults dual influence of digitalization: opportunities and challenges

In contemporary Europe, with the increasing revolution in information technologies, young adults are becoming politically connected to digital politics rather than traditional political attitudes. The digital agora facilitates their new political attitudes, which created an interactive electoral hall for young people to express their political yearnings and aspirations. This has helped reduce barriers to entry for new movements and introduced new logics of collective action. This digital mobilization approach that facilitates digital politics is less about top-down, but more about decentralized assembly free from rigorous leadership control that allow for independent networks that can form rapidly around shared identities or grievances. Digitalization has thus facilitated online petitions, virtual campaigns, and live political exchanges. Young adults may increasingly engage in politics remotely, including signing petitions and joining internet advocacy organizations.

In modern days, political digitalization has created a novel pathway for political engagement that resonates with the youth's preferences for effective political participation. Today, social media platforms have lowered barriers to political entry as they allow for easy consumption and dissemination of political information among community members. Considering the massive internet connectivity and social media usage among the young adults in Europe, digital politics has become much easier across the continent, for instance in 2024, 97% of those aged 16–29 in the EU reported daily internet usage. The digital world has helped in increasing their political activities across the continent. Eurostat reports that in 2024, individuals aged 16 to 29 were, on average, more inclined to voice their opinions or engage in online consultations for civic or political matters via the internet compared to the whole population. In 2024, online civic or political engagement among youth was most prevalent in Slovenia, where 51% of individuals aged 16–29 voiced an opinion or participated in activities related to civic or political life, followed by Greece at 37% and Italy at 35% ( Eurostat, 2025).

Activist youth across Europe and the world have relied heavily on digital technologies. The "Fridays for Future" strike, started by a 19-year-old girl from Sweden named Greta Thunberg, helped bring attention to important social issues and encouraged other young people to take action for a better world ( Murugan *et al*., 2023: 97–100). What began in August 2018 with a small, attractive girl in front of the Swedish Parliament quickly gained traction across Europe (particularly in Austria, Belgium, the Netherlands, Germany, Finland, Denmark, and Poland) and the rest of the world (particularly in North America and Australia) thanks to the extensive use of social media. In different nations, like the UK, Ireland, and Germany, young people are taking action on climate change through online means. In the UK, for example, students and youth demand a lower voting age of 16 to have a say in public elections that benefit their interests ( NYCI, 2025). This campaing is very effective online, further justifying the assumption of the relevance of digitalization on youth political participation.

Through the use of social media, the internet has made it possible for young adults to communicate directly with politicians or institutions. This has resulted in the development of a sense of accountability that is independent of the gatekeepers of traditional media environments. These days, political parties and politicians are more likely to have social media profiles and participate in online political activities than their predecessors were. Such digital leaders include Beppe Grillo, who influenced the path of Italian politics, Giorgia Meloni, the Prime Minister of Italy, has garnered attention on social media, alongside local politicians such as former Barcelona mayor Ada Colau, who have been identified as "influencer-politicians" for their extensive use of social media, particularly TikTok, and their youthful communication style during her recent term, Volodymyr Zelensky, a former comedian, adeptly utilized the internet realm to further his political career, far-right leaders such as Marine Le Pen, Matteo Salvini, and Eric Zemmour have utilized Tik-Tok to disseminate not only negative or fear-inducing messages but also more personal content, thereby adapting to the platform's style, a strategy that aids in engaging like-minded youth in political activities ( Villaplana & Fitzpatrick, 2024: 03–04). This has created a more digital political connectivity between the leaders and followers, especially the youth. Across Europe today, considering the high level of internet usage accentuated by the widespread internet coverage compared to some regions in the developing world, European youth have more chances of engaging in digital politics. Realizing that they can hold their leaders accountable via signing online petitions and calling leaders to action by expressing their opinions on social media platforms, young European adults became more motivated in politics. These young citizens recognize that the digital technology of the printing press and continuous engagement with reading empowered the public to critique the government and hold their representatives accountable; consequently, they are inspired by contemporary political technology to enhance their involvement in political processes ( Loader, 2007: 9). Young individuals are inclined to engage in political activities, particularly when motivated by their peers through social media. Contemporary youth in European politics find it far more appealing to endorse an online petition, shared by a friend, about internet surveillance, rather than actively supporting the comprehensive agenda of a hierarchical entity such as a political party ( Sloam, 2014: 218). Comparatively, young adults are more likely to participate in digital politics, such as signing online petitions on a particular issue and internet activism, than older cohorts ( Sloam, 2016: 524). Thus, the digital world influences young adults' civic functions.

Social media also motivates young adults to participate in politics actively, realizing they can help in community building and support human and animal rights through digital politics. Digital platforms enable marginalized youth, particularly ethnic minorities, to discover community, express counter-narratives, and advocate for their rights. The youth are inclined to participate more in politics, believing it will positively impact their socioeconomic life and subsequent community development ( Koc-Michalska *et al.*, 2016: 1807). The youth now realize they can call their leaders' attention to addressing nagging issues affecting their society through social media campaigns, solving problems that contribute to societal development. The youth may do this via the concept of "civocracy," which transforms digital civic participation by offering a safe and inclusive platform for citizens to engage in decision-making processes actively. Integrating online tools with offline strategies motivates young adults to engage actively in their communities. This can help in bridging the divide between governments, NGOs, and communities, and promoting substantive dialogue and collaboration among youth and other societal stakeholders ( Lesaint, 2025).

One major factor that also influence the youth’s political digitalization and motivates their civil engagement is the need to take part in counter-narratives against hate speech and misinformation that is often being spread in the digital environment ( Poole & Giraud, 2019: 2). Young adults in Europe are influence by the digital narratives on the media regarding the continent especially on issues of xenophobia and anti-immigration rhetorics, which make them willing to engage in digital world to counter such narratives the right way. Since there are specific anti-immigrant issues, there are also numerous pro-immigrant campaigns across the continent that the mainstream media hardly broadcasts. Currently, there is minimal discourse regarding the activities of immigration solidarity organizations, including France-based associations such as Gisti, Fasti, and Migreurop, the UK-registered charity Migrants’ Rights Network (MRN), No Borders, No One Is Illegal (NOII), Boats 4 People (B4P), a solidarity flotilla in the Sicilian canal, and When You Do not Exist (WYDE), all of which advocate for the human rights of migrants, refugees, and asylum-seekers in Europe and at its borders ( Cantat, 2016: 9). Several young adults across Europe participate or take part in the activities of this organization via social media to assist in the changing narratives of anti-immigrants’ rhetoric some associate some European countries with. By so doing, they not only help desensitization but also, as a matter of civic participation, contribute to the European integration project as a matter of civic participation.

### Digitalization: challenges and threats to meaningful participation

Although the instruments of digitalization facilitate the cultivation of political consciousness among youth, they also present considerable problems. This encompasses the proliferation of hate speech, misinformation, cyberbullying, and foreign meddling in electoral processes. Therefore, the digital arena is associated with numerous perils that can negatively affect the quality and equality of civic engagement in society.

A significant challenge for digital politics in most societies is the persistent digital divide resulting from both the first-level divide, internet accessibility, and the second-level divide, possessing the usage skills of the digital platforms. These two divides are some of the shortcomings of digital politics among the youth in the contemporary world. In some regions, while there is a lack of access to the internet, which automatically eliminates the youth from the digital political world, in some societies, most youth lack the knowledge on how to use the digital instruments, exceptionally the emerging artificial intelligence to support their civic engagement, is lacking. These two divides predominantly impact socioeconomically disadvantaged adolescents, since their insufficient resources may hinder their digital literacy, impairing their ability to connect with or critically assess online political content ( Hargittai, 2002: 2-4). These digital divides are more visible in lower economic development. In these countries, regions that are smaller and more prosperous societies are easier to connect with the digital world, have a high level of e-governance, e-voting, e-health, and e-commerce usage than those that are bigger and poorer ( Cruz-Jesus *et al.*, 2012: 280). Digital divide in Europe has not only affected digital politics but also reflects significant disparities in access, usage, and skills between and within the countries in the continent. Researchers and scholars continue to debate a North-Western and South-Eastern divide, jeopardizing the EU's cohesion objectives. A typical example was that in 2022, 95% of Danish homes possessed fixed extremely high-capacity network coverage, whilst Greece lagged at 62% ( European Commission, 2022). 

Disparity or differences in internet usage that emanate from the level of digital literacy and investment in human capital have further widened the digital usage and skills gap between vital countries like Finland and the least developed nations. For instance, Small and medium-sized enterprises (SMEs) in the Netherlands and Sweden are much more advanced in integrating artificial intelligence (AI) and cloud technology, according to research conducted in 2023. There is also a significant disparity in e-commerce; research shows that in terms of the percentage of businesses participating in e-commerce, Denmark, Ireland, and Sweden have the largest proportion. As a further point of interest, businesses from Ireland, the Czech Republic, and Belgium have the highest turnover from activities related to web commerce. On the other hand, Bulgaria and Romania have a relatively low level of company participation in e-commerce for both variables. Both of these nations, along with Cyprus, Greece, and Latvia, have the lowest turnover from eCommerce in the European Union ( Hunady *et al*., 2022: 43) These disparities create issues that feed digital inequality among young adult in the continent, which further affect the chances of some of them to actively participate in digital politics like their peers across the continent.

The rising polarization and partisan and ideological echo chambers also hamper the significant positive effects of digital politics. More than their counterparts in the United States, young adults found themselves across Europe polarized ( Hobolt *et al.*, 2024: 1465). These can amplify ideological segregation that can be detrimental to the harmonious political space on the continent. Comparative research reveals a larger clustering in countries where party systems are divided and partisan media is prominent. For example, retweet homophily is more prevalent in Poland and Hungary than in Germany or the Netherlands, where public-service media and cross-cutting exposure are more prevalent ( Matuszewski & Szabó, 2019, pp. 4–5). As a result of this polarization, a homogenous information environment is being created, which serves to reinforce preexisting opinions and restricts its exposure to a variety of perspectives, so making civic engagement more difficult.

Increasing digitalization makes the young adults very susceptible to the danger of online news, making them consumers of sophisticated disinformation campaigns that can hamper the correct political perception, or change it with wrong perception by eroding trust in the minds of the youth and negating the ideal democratic institutions in an established society institution ( European Commission, 2022). Fake news and disinformation creation have become a serious concern for many countries' policy makers considering the danger they pose to society's political and social setting ( Fletcher *et al*., 2018: 1-2). Today, even in Spain, Italy, Greece, Germany, Denmark, and Sweden, there is increasing misinformation and concern about the distrust of political information on social media, which is the environment of digital politics ( Rodríguez-Pérez *et al*., 2024). Most youth who realize the existence of fake news become disinterested in digital platforms, subsequently affecting their willingness to participate in political activities via digitalization. Misinformation is an enemy of digital politics as it discourages young people from being active in online political activities for fear of being misinformed.

Slacktivism, generally defined as supporting a political or social cause through minimal efforts or the ease of online support-such as social media or petitions, can undermine active political engagement, particularly among youth often engrossed in internet activities. Slacktivism makes the youth very active in their online political environment, so they are reluctant to leave their comfort zone to participate in more rigorous political functions. In other words, slacktivism offers low-risk and low-cost engagement, which some critics see as substituting more impactful offline political activities ( Kristofferson *et al*., 2020: 1149–52).

## Conclusion

Due to the fact that technology acts as a force that equalizes most societies, digitalization has become increasingly important. A democratization of access to education, employment opportunities, and business opportunities is what makes the notion a reality. The fact that this issue got more severe during the COVID-19 epidemic is perhaps an indication that digital connectivity became more and more important for political and economic participation. At the same time, physical encounters were restricted, rendering individuals without access more susceptible to political and financial marginalization ( Paskaleva, 2025: 125). Digitalization has a considerable impact on the political engagement of young people in modern Europe for a number of reasons, including the modification of participation tactics and the enhancement of accessibility. As a consequence of this transition, new forms of political action have emerged, and the traditional definition of political involvement has been redefined. As a result, the political environment that young people are exposed to has become more dynamic and responsive. Digital platforms enable young people to rapidly organize and mobilize for issues and events they care about, including environmental activism via movements like Fridays for Future. Through social media, the youth across Europe and even in other parts of the world participate actively in climate issue-based campaigns, which drew the attention of policymakers in their society.

Political debate finds venues on social media and online communities, allowing young people to freely express ideas and interact with political material. Today, young adults in Europe who are politically engaged have integrated social media into their political activities, and most of them, without digital connectivity, have not done so ( Vromen *et al.*, 2015: 80–3). Research in countries such as Poland, the Netherlands, and Italy shows that young people are eager to participate in politics when provided with suitable tools. These tools include e-democracy platforms which hold remarkable potential to promote e-participation among young citizens ( Ambrosino *et al*., 2023). E-democracy projects offer customized tools that appeal to the tastes of young people, so boosting their eagerness to take part in political events

Despite the influence of digitalization on young European citizens in political participation, the idea of increasing or relying solely on the internet for civic duties is associated with numerous challenges, barriers, and obstacles. For instance, obstacles, including digital inequality and safety issues, can hamper participation. Concerns about the invasion of their privacy may cause young people to be reluctant to take part in some political activities that take place online. Furthermore, the digital gap that exists between Eastern and Western Europe has an effect on the level of political involvement ( Simionov *et al*., 2023). This is because varied access to digital resources has an effect on participation rates. From these, we can conclude that although digitalization gives young people great opportunities for political involvement, it also presents issues that must be resolved to guarantee fair involvement among many groups and areas. For these reasons, we recommend the following:

To harness the positive potential of digitalization towards digital democratic development across the continent, policymakers and educators should focus on:

Media and information literacy should be integrated into the education system at all levels of education to equip society from primary to tertiary institutions to navigate the online ecosystem. This approach should also be applied in informal educational settings to include the larger society in digital literacy programs. The essence of the digital education program should not only be about teaching digital knowledge. However, it should also enlighten young people about the dangers of negativity, such as fake news, internet theft, and other vices that the contemporary digital world encompasses. Governments should also partner with Civil Society Organizations in order to eradicate digital divides by providing affordable and accessible internet to all local communities.

Digital accountability and transparency should also be a priority for policymakers in making digitalization a friendly environment for citizens. Regulatory frameworks via EU-wide policies like the Digital Services Act (DSA) should be strengthened to function effectively, especially in holding digital platforms accountable for curbing societal disinformation. This act can also serve to checkmate polarization, which hinders democratic processes. Within the continent, there is a need for policymakers and institutions such as political parties to innovate and redesign online engagement for the better in order to provide an avenue for informal decision-making processes that would bridge the gap between digital activism and institutional politics ( Dubois & Blank, 2018: 732). Since southern and center European countries, like Poland and Italy, show more ideological grouping online than their northern European counterparts, like Denmark, their institutions should lead the way in addressing this problem ( Koc-Michalska *et al*., 2024: 2–3). Finally, the future of European democracy hinges not on restricting digitalization, but on proactively managing its dangers while enhancing its capacity to involve the upcoming generation of citizens.

## Ethics and consent

Ethical approval and consent were not required

## Data Availability

The article highlights the advancement of democratic technology in shaping political participation among yoounfg adult in contemporary Europe. It defend the arguement that Unlike traditional political parties, digital parties utilize digitalization to create accessible structures that promote broader and more effective participation among youth and the general public. Through reviewing of 30 scholarly articles published over the past decade on the Web of Science platform, the article explore how digital technologies foster political enthusiasm across Europe. It justified that technologized processes such as e-democracy, e-elections, e-governance, and e-participation offer customized tools that appeal to younger demographics, thereby increasing their willingness to engage in political activities. The future publication will focus on the projected influence technology could have in the democratic practice in European society. The dataset, gathering quantitative information and metadata, is made available by the authors in line with the requirement of the framework of the Open Access to JRC Research Infrastructures. The complete dataset can be downloaded at https://doi.org/10.5281/zenodo.17444098 under a CC BY 4.0 license. Wara (2025). Influence of Digitalization on Political Participation among Young Adults in Contemporary Europe. 29th Annual Conference of Central European Political Science Association (CEPSA) Politicization and Post-politics in Times of New Challenges 18–19 September 2025, University of Warsaw, Poland (CEPSA), University of Warsaw, Poland. *Zenodo*. https://doi.org/10.5281/zenodo.17444098
