# Supplementary material for: Influence of digitalization on political participation among young adults in contemporary Europe
Source: Open Res Eur. 2026 Apr 16;5:360. Originally published 2025 Nov 26. [Version 2] doi: 10.12688/openreseurope.21521.2 (PMC13153763; doi:10.12688/openreseurope.21521.2)
Supplement: Peer review history

# Reviewer report: Reviewer response for version 2

**DOI:** 10.21956/openreseurope.25149.r72441

**Published:** 2026-05-09

**Authors:** Deni Zein Tarsidi

**Affiliations:** 1 Universitas Sebelas Maret Surakarta, Surakarta, Surakarta, Indonesia

The revised manuscript shows clear improvement compared to the previous version. The addition of the section “Digitalization and Political Participation in Modern Europe” helps strengthen the conceptual discussion and makes the overall argument more coherent. The inclusion of the PRISMA flowchart, the table of reviewed studies, and the expanded methodological explanation also improves the transparency of the study.

 The decision to focus more consistently on “political participation” rather than using broader terms such as “civic engagement” is also appropriate, as it reduces conceptual ambiguity throughout the paper.

 At the same time, several points still need further attention.

 The main concern relates to the methodological positioning of the study. The article describes the research as a qualitative meta-synthesis, yet many of the studies included in the review are quantitative, survey-based, or statistical in nature. This does not necessarily weaken the study, but the manuscript should explain more clearly how these different types of studies are integrated within the interpretive meta-synthesis framework.

 In addition, some sections remain descriptive rather than analytical. The paper successfully summarizes existing discussions on digital political participation, but the theoretical contribution could still be developed further. A deeper engagement with recent debates on digital democracy, algorithmic influence, or platform politics would strengthen the analytical depth of the article.

 There are also a number of grammatical and stylistic issues across the manuscript. Some sentences are repetitive or overly long, which affects readability in several sections. A careful language revision is recommended.

 Overall, the revised version is considerably stronger and addresses many of the concerns raised previously. The article discusses an important and timely issue, particularly in relation to youth political participation and digital transformation in Europe. With additional clarification of the methodology and further refinement of the analysis, the manuscript would make a useful contribution to the field.

If applicable, is the statistical analysis and its interpretation appropriate?

Not applicable

Is the study design appropriate and is the work technically sound?

Partly

Is the work clearly and accurately presented and does it engage with the current literature?

Partly

Are the conclusions drawn adequately supported by the results?

Partly

Are sufficient details of methods and analysis provided to allow replication by others?

No

Are all the source data and materials underlying the results available?

Partly

Reviewer Expertise:

My expertise is in civic education/citizenship studies, civic engagement, digital citizenship and youth political participation, and digital media literacy. I can assess the conceptual framing, literature engagement, methodological transparency, and the appropriateness of statistical analyses and their interpretation where applicable.

I confirm that I have read this submission and believe that I have an appropriate level of expertise to confirm that it is of an acceptable scientific standard, however I have significant reservations, as outlined above.

# Reviewer report: Reviewer response for version 2

**DOI:** 10.21956/openreseurope.25149.r72442

**Published:** 2026-05-07

**Authors:** Gianfranco Polizzi

**Affiliations:** 1 University of Birmingham, Birmingham, England, UK

The paper is in better shape, and the analytical procedures are now clearer. There are still, however, several points that require further clarification and development:

- “Step 1” – which gap is being referred to? The author should be more precise about what this gap entails.
- The term “influence” in the research question is misleading, as it is more appropriate for a quantitative meta‑analysis concerned with causality or intervention effectiveness. A term such as “shape” may be more suitable.
- Some studies in Table 1 are quantitative, even though the meta‑analysis focuses solely on qualitative research. Were these mixed‑methods studies, and was the qualitative component extracted specifically for this review?
- The themes presented appear to reflect the main arguments of the reviewed papers, but they do not constitute a synthesis of qualitative findings (e.g., quotations, codes and themes).
- If the sections on mobilisation and digitalisation are not part of the findings, they may be better placed in the literature review. This section could also be renamed to avoid confusion with the paper’s aim of reviewing relevant literature.
- Some paragraphs are under‑referenced – for example, the second paragraph in the first subsection under “Results.”
- A table summarising the main findings would help visualise the key themes emerging from the literature.

If applicable, is the statistical analysis and its interpretation appropriate?

Not applicable

Is the study design appropriate and is the work technically sound?

No

Is the work clearly and accurately presented and does it engage with the current literature?

No

Are the conclusions drawn adequately supported by the results?

No

Are sufficient details of methods and analysis provided to allow replication by others?

No

Are all the source data and materials underlying the results available?

No

Reviewer Expertise:

media and information literacy, civic engagement, political participation, digital citizenship

I confirm that I have read this submission and believe that I have an appropriate level of expertise to confirm that it is of an acceptable scientific standard, however I have significant reservations, as outlined above.

# Reviewer report: Reviewer response for version 2

**DOI:** 10.21956/openreseurope.25149.r72443

**Published:** 2026-04-17

**Authors:** Christian Fuchs

**Affiliations:** 1 Paderborn University, Germany, Paderborn, Germany

The revision of the paper has improved it and has taken my points into account, so I am happy to now approve it.

If applicable, is the statistical analysis and its interpretation appropriate?

Not applicable

Is the study design appropriate and is the work technically sound?

Yes

Is the work clearly and accurately presented and does it engage with the current literature?

Yes

Are the conclusions drawn adequately supported by the results?

Partly

Are sufficient details of methods and analysis provided to allow replication by others?

No

Are all the source data and materials underlying the results available?

Partly

Reviewer Expertise:

Digital Media and Society

I confirm that I have read this submission and believe that I have an appropriate level of expertise to confirm that it is of an acceptable scientific standard.

# Reviewer report: Reviewer response for version 1

**DOI:** 10.21956/openreseurope.23279.r65540

**Published:** 2026-01-30

**Authors:** Gianfranco Polizzi

**Affiliations:** 1 University of Birmingham, Birmingham, England, UK

Thank you for the opportunity to review this manuscript. The study presents findings from a meta‑synthesis of 30 scholarly articles published between 2014 and 2025, aiming to examine how digitalisation influences political participation among young adults in Europe. While the topic is timely and the manuscript is generally well‑written, the article contains several substantive issues that require a full rewrite rather than substantial revisions.

- Although this is a meta‑synthesis of existing research, the analysis lacks a more integrated and interpretive account of the key findings. At present, the contribution feels largely descriptive. The author could strengthen the manuscript by developing a conceptual model that explains how digitalisation shapes political participation in Europe, including both opportunities and risks emerging from online environments.
- Concepts like civic engagement and political participation are used interchangeably, yet they have important conceptual distinctions. These differences should be clearly articulated to avoid conceptual ambiguity.
- The author states that they conducted a meta‑ethnography (Noblit & Hare), but the rationale for excluding quantitative studies is not sufficiently explained. The methods section is also too brief. More detail is needed regarding the analytical procedures. What specific approach was used to synthesise the qualitative findings? How were themes generated and refined? Was the quality of the included studies appraised, and if so how?
- The manuscript should include details about the number of papers identified, screened, excluded and ultimately included. A flow diagram similar to a PRISMA chart would greatly improve transparency. Additionally, a table summarising the characteristics of the included studies is essential for readers to assess the scope and quality of the evidence base.
- The choice of mobilisation theory requires clearer justification. It is not evident why this particular framework was selected over other relevant theories. Relatedly, the author should clarify position this framework in relation to the broader literature and more clearly discuss both institutional and non‑institutional forms of political participation.
- The results section lacks illustrative evidence from the reviewed studies. A meta‑ethnography requires systematic engagement with qualitative findings, including the use of exemplar quotes or detailed descriptions from the primary studies. Currently, the analysis appears insufficiently grounded in the data and lacks methodological rigour.
- The conclusion introduces terms (e.g., media and information literacy) that are not sufficiently defined or discussed earlier in the manuscript. Also, the discussion should be strengthened by further elaborating on the policy and practical implications of the findings. A section outlining the limitations of the study is also needed.

If applicable, is the statistical analysis and its interpretation appropriate?

Not applicable

Is the study design appropriate and is the work technically sound?

No

Is the work clearly and accurately presented and does it engage with the current literature?

No

Are the conclusions drawn adequately supported by the results?

No

Are sufficient details of methods and analysis provided to allow replication by others?

No

Are all the source data and materials underlying the results available?

No

Reviewer Expertise:

media and information literacy, civic engagement, political participation, digital citizenship

I confirm that I have read this submission and believe that I have an appropriate level of expertise to state that I do not consider it to be of an acceptable scientific standard, for reasons outlined above.

# Reviewer report: Reviewer response for version 1

**DOI:** 10.21956/openreseurope.23279.r68591

**Published:** 2026-01-28

**Authors:** Christian Fuchs

**Affiliations:** 1 Paderborn University, Germany, Paderborn, Germany

Review

 This article analyses how digitalization has transformed political participation among young adults in contemporary Europe.

 The article is well written and well-grounded in relevant literature. It addresses a question that is important to the study of digital politics today.

 The literature review in the background section is well written. It presents works that have analysed digital participation of young people in Europe.

 The paper uses a qualitative meta-analysis of relevant research conducted about young adults’ and digital participation in Europe. The “Method” section explains the paper’s methodology.

 The selection of relevant articles has been explained in a feasible manner:

 “In terms of data collection, a thorough literature review was performed utilizing the Web of Science database. The investigation encompassed the timeframe from 2014 to 2025 and exclusively featured peer-reviewed academic publications authored in English. Search keywords encompassed combinations of digitalization, youth, political engagement, Europe, e-democracy, and online activism. The inclusion criteria mandated that each study (a) concentrate on European contexts, (b) examine digital tools or platforms concerning political engagement, and (c) encompass youth populatons aged 15–35.“

 What I think would be helpful in the methodology section is a table that gives an overview of all 30 papers that were identified, giving an ID to each, and mentioning the following information for each paper in a separate column:

 ID (1, 2, … 30)

 Authors

 Publication year

 Journal title

 Main research question

 Methodology

 Number of citations thus far in Web of Science 

 Date of access (Web of Science)

 This would help giving the reader an immediate overview of what papers were included in the meta-analysis.

 The “Results” section presents the results of the analysis. The presentation is insightful and well written. However, I was not always sure what statements were derived from the 30 papers and which one from other works (e.g. Eurostat 2025).

 I recommend that more references are added to the results section and that the author uses paper IDs (1, 2, … 30) that refer to a table papers included in the meta-analysis. It then becomes evident to the reader what information in the results section has been derived from what papers included in the meta-analysis. Furthermore, I think it would be advantageous to indicate what information has been derived from more than one paper. Recurrent findings that we can find in many papers is the most relevant insights one can gain from a meta-analysis.

 Conclusion: The results have been well summarised. I do, however, think that the author could more pinpoint the most central findings by adding a list of 3-5 bullet points that summarise the main findings about young people’s digital political participation in Europe that were recurrent in the papers included in the meta-analysis.

 Overall, the findings are interesting and the paper is well written. They can be presented in a better way than at the moment.

 My recommendation is:

 Approve with Reservations

 Christian Fuchs

If applicable, is the statistical analysis and its interpretation appropriate?

Not applicable

Is the study design appropriate and is the work technically sound?

Yes

Is the work clearly and accurately presented and does it engage with the current literature?

Yes

Are the conclusions drawn adequately supported by the results?

Partly

Are sufficient details of methods and analysis provided to allow replication by others?

No

Are all the source data and materials underlying the results available?

Partly

Reviewer Expertise:

Digital Media and Society

I confirm that I have read this submission and believe that I have an appropriate level of expertise to confirm that it is of an acceptable scientific standard, however I have significant reservations, as outlined above.

# Reviewer report: Reviewer response for version 1

**DOI:** 10.21956/openreseurope.23279.r65545

**Published:** 2026-01-06

**Authors:** Deni Zein Tarsidi

**Affiliations:** 1 Universitas Sebelas Maret Surakarta, Surakarta, Surakarta, Indonesia

The manuscript tackles a question that genuinely matters right now: how digitalization shapes political participation among young adults in contemporary Europe. A synthesis on this topic is potentially valuable given how broad and fragmented the literature is. The central takeaway that digitalization can expand opportunities for engagement while also amplifying risks such as inequality, polarization, misinformation, and *slacktivism *is sensible and broadly in line with what readers might anticipate.

 That said, the manuscript currently reads closer to a well-informed narrative review than a transparent meta-synthesis. The themes are plausible, but the pathway from the included studies to the final conclusions is not consistently visible. For that reason, I consider the conclusions to be only partly supported by the results as presented.

 To make the article scientifically sound, the review process needs to be more traceable and replicable (exact search strings and dates, clear screening procedures, PRISMA-style flow and reasons for exclusions). The paper should also show what was actually included via a study-characteristics table and map key themes to the specific studies that support them, alongside at least a brief quality appraisal of included studies. Conceptual boundaries should be tightened (definitions of digitalization and political participation; justification of the 15–35 age range), and the data availability statement should be clarified to reflect the review materials. With these revisions (plus language editing and a short limitations section), I would recommend Approved with reservations.

If applicable, is the statistical analysis and its interpretation appropriate?

Not applicable

Is the study design appropriate and is the work technically sound?

Partly

Is the work clearly and accurately presented and does it engage with the current literature?

Partly

Are the conclusions drawn adequately supported by the results?

Partly

Are sufficient details of methods and analysis provided to allow replication by others?

No

Are all the source data and materials underlying the results available?

Partly

Reviewer Expertise:

My expertise is in civic education/citizenship studies, civic engagement, digital citizenship and youth political participation, and digital media literacy. I can assess the conceptual framing, literature engagement, methodological transparency, and the appropriateness of statistical analyses and their interpretation where applicable.

I confirm that I have read this submission and believe that I have an appropriate level of expertise to confirm that it is of an acceptable scientific standard, however I have significant reservations, as outlined above.
